# Supplementary material for: The microbiology of periprosthetic joint infections as revealed by sonicate cultures in Korea: Routine use of fungal and mycobacterial cultures is necessary?
Source: PLoS One. 2024 Aug 15;19(8):e0309046. doi: 10.1371/journal.pone.0309046 (PMC11326641; doi:10.1371/journal.pone.0309046)
Supplement: S1 Table — (DOCX) [file pone.0309046.s001.docx]

**Supplementary Table 1.** Diagnostic criteria for hip or knee periprosthetic joint infection.

| Modified Clinical Criteria | | |
| --- | --- | --- |
| Sinus tract shows evidence of communication with the joint, or visualization of the prosthesis.  Visual purulence around prostheses in intraoperative findings. Positive histology (≥ 5 neutrophils in ≥ 5 HPF). | | |
| 2021 European Bone and Joint Infection Society (EBJIS) Criteria | | |
| One of six confirmatory criteria | or | Two suggestive criteria: “Infection likely” |
| Sinus tract in communication with the joint, or visualization of the prosthesis, |  | Relevant clinical features or elevated CRP level (> 1mg/dl), |
|  |  | Elevated synovial WBC count (> 1,500) or PMN (> 65%), |
| Elevated synovial WBC count (> 3,000) or PMN (> 80%) |  | Positive synovial culture, |
| Positive alpha-defensin test, |  | Single positive culture of either intraoperative fluid or tissue, |
| At least two culture samples positive for the same microorganism, |  | Sonication : > 1 CFU/mL of any organism, |
| Sonication^*^ : > 50 CFU/mL of any organism (> 200 CFU/mL if centrifuge applied), |  | Positive histology (≥ 5 neutrophils in a single HPF), |
| Positive histology (≥ 5 neutrophils in ≥ 5 HPF). |  | Positive WBC scintigraphy. |

PJI, Periprosthetic Joint Infection; CFU, colony-forming unit; CRP, C-reactive protein; HPF, high-power field; PMN, polymorphonuclear neutrophils; WBC, white blood cell.

^*^ If other variations to the protocol are used, the published cut-offs for each protocol must be applied. In this study, we used the Mayo Clinic protocol and considered 20 CFU/10ml as the validated cutoff value.
