# Supplementary material for: The microbiology of periprosthetic joint infections as revealed by sonicate cultures in Korea: Routine use of fungal and mycobacterial cultures is necessary?
Source: PLoS One. 2024 Aug 15;19(8):e0309046. doi: 10.1371/journal.pone.0309046 (PMC11326641; doi:10.1371/journal.pone.0309046)
Supplement: Attachment — Submitted filename: Responses_to_Reviewers_20240802.docx [file pone.0309046.s003.docx]

Title: The microbiology of periprosthetic joint infections as revealed by sonicate cultures in Korea: Routine use of fungal and mycobacterial cultures is necessary?

Dear Editor-in-Chief

I would like to thank you for giving us the opportunity to revise our work. We have revised the manuscript in light of the valuable suggestions, and we believe it has been greatly improved. Attached you will find our revised manuscript, as well as our responses to the comments. This manuscript has not been published in any journal and is not under consideration for publication elsewhere. All authors including myself have seen and approved this manuscript.

Again, we thank you and the reviewers for a constructive and helpful review.

Here are the detailed descriptions

Sincerely yours,

Kyung-Hwa Park

Department of Infectious diseases,

Chonnam National University Medical School

42 Jebong-ro, Dong-gu, Gwangju, 61469, Korea

Responses to edditor’s additional comments:

#1: This is an important topic especially since we need a diverse voice in Ortho research. However, it needs further work before being ready for publication.

- As recommended, we have added a statistical analysis section in the M&M.

#2: Separate the conclusion section.

- As recommended, we have separated the conclusion section.
